# Primary Bone Lymphoma of the Shaft of the Tibia, Mimicking Subacute Osteomyelitis

**DOI:** 10.7759/cureus.38070

**Published:** 2023-04-24

**Authors:** Prabhakaran Jayaprakasan, Arun Warrier

**Affiliations:** 1 Orthopedics and Traumatology, NMC Specialty Hospital, Abu Dhabi, ARE; 2 Medical Oncology, Aster Medcity, Cochin, IND

**Keywords:** ct-guided biopsy, misdiagnosis, radiotherapy, chemotherapy, immunohistochemistry and biopsy, subacute osteomyelitis, primary bone lymphoma

## Abstract

We present the case of a 32-year-old healthy male who presented with a three-month history of insidious onset pain and swelling over the right tibia. Initial radiographs and imaging pointed to a diagnosis of subacute osteomyelitis, as there was no cortical destruction, periosteal reaction, or soft tissue involvement. The patient underwent surgery for osteomyelitis. However, the histopathology and immunohistochemistry (IHC) findings pointed to a possible B-cell lymphoma diagnosis. The patient was referred to a tertiary-level oncology centre, where a repeat biopsy and positron emission tomography (PET) scan confirmed a diagnosis of primary bone lymphoma (PBL). Treatment was initiated immediately in the form of a combination of chemotherapy and radiotherapy, and the progress was followed up with further scans at four-month intervals. The patient achieved remission nine months after the initiation of treatment.

## Introduction

Primary bone lymphoma (PBL), a subtype of non-Hodgkin’s lymphoma, is defined as either development in a single bone with or without regional lymph node involvement or multiple bone lesions without visceral or lymph node involvement [1]. Patients with other visceral site involvement or multiple lymph nodes at multiple sites, in addition to a bone tumour, are excluded from PBL. It accounts for 7% of primary bone tumours, 4%-5% of extra-nodal lymphomas, and less than 2% of all lymphomas in adults [2].

Oberling, in 1928, first described PBL. Parker and Jackson reported a series of cases under the designation ‘reticulum cell sarcoma of the bone’ and distinguished PBL as a distinct entity [3]. Immunohistochemistry (IHC) studies later confirmed the B-cell and lymphoid origins of PBL. The disease shows a slightly higher male preponderance.

There is a higher predilection for the disease to occur in the axial skeleton as compared to the appendicular skeleton, with the femur being involved in nearly a third of the cases [4]. Spread to lymph nodes and bone marrow occurs in about 28% and 35% of cases, respectively.

The diagnosis of PBL is based on a combination of clinical features, radiographic findings, and histopathology, of which histopathology is the most definitive. On microscopy, tumour cells typically appear consistent with follicular centre or centroblastic cell types and are fairly large in size [5]. Flow cytometry demonstrates immunoreactivity for B-cell markers including CD45, CD20, CD21, and CD79a, with variable immunoreactivity for CD75 and CD10 [6].

## Case presentation

A 32-year-old male presented with a history of right shin pain of three months’ duration with intermittent low-grade fever. There was no past history of trauma, infections, contact with tuberculosis, or any major illness. On examination, he had diffuse swelling over the proximal to middle third of the tibia, with normal overlying skin, tenderness on deep palpation, and a local rise in temperature. There were no sinuses, adjacent joint involvement, or regional lymph node enlargement.

**Figure 1:**
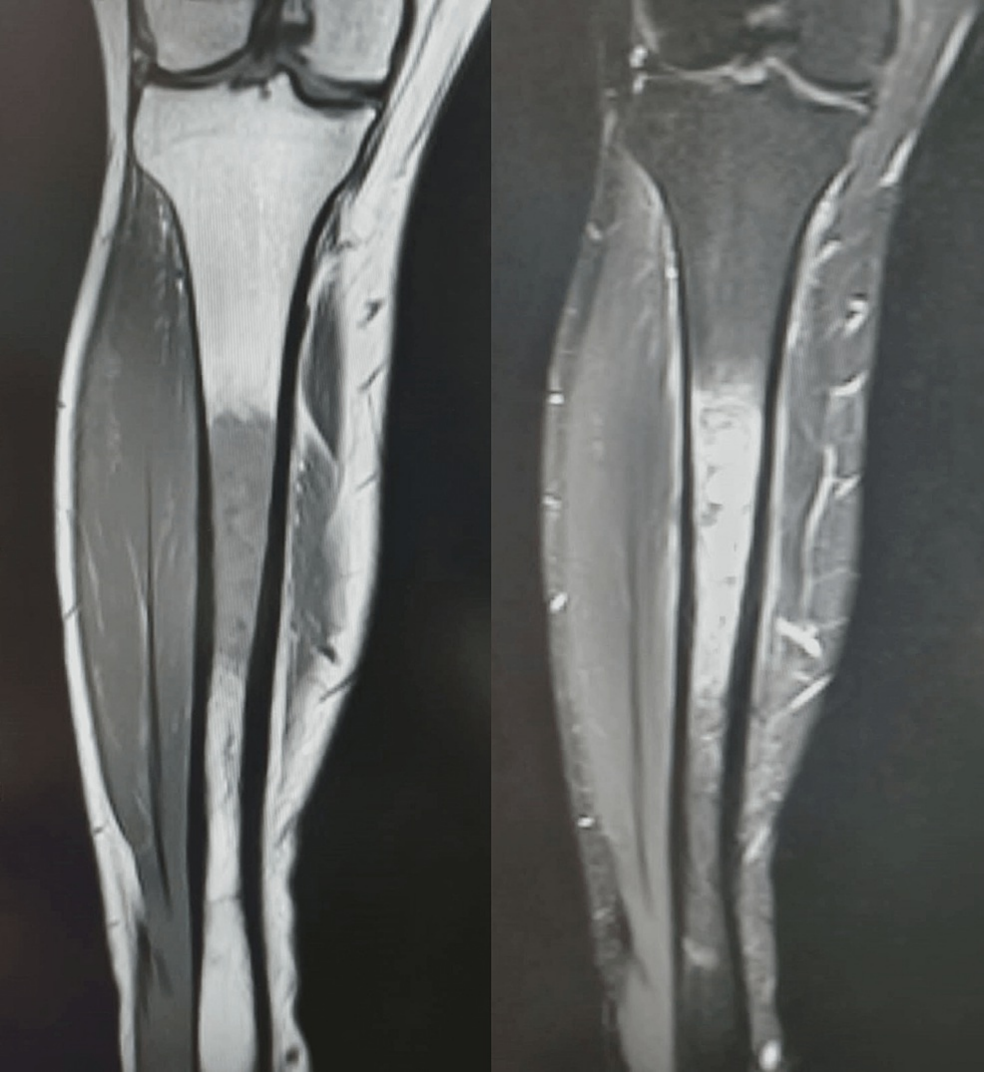
T1 and STIR coronal images

He was treated symptomatically at another centre for six weeks without resolution of pain. Baseline blood investigations and radiography were normal. A magnetic resonance image (MRI) scan (Figure 1) with contrast reported a sharply defined heterogenous area of altered marrow enhancement in the midshaft tibia, approximately 12 cm in length, with minimal oedema noted in the surrounding soft tissues. There was no evidence of a cortical break, periosteal reaction, or abnormal soft tissue signals. The rest of the tibia appeared normal. The findings were suggestive of osteomyelitis of the tibia, and a clinical diagnosis of subacute osteomyelitis of the tibia was considered.

He underwent surgery with curettage of the lesion through a proximal tibial approach, reaming, irrigation, aspiration, and local antibiotic delivery. Samples were sent for culture, sensitivity, and histopathological examination (HPE). The reaming was done all the way to the distal tibia. Two grams of vancomycin in twenty grams of calcium phosphate bone cement were inserted under image intensifier control at the area of the suspected osteomyelitis for local antibiotic effect.

**Figure 2:**
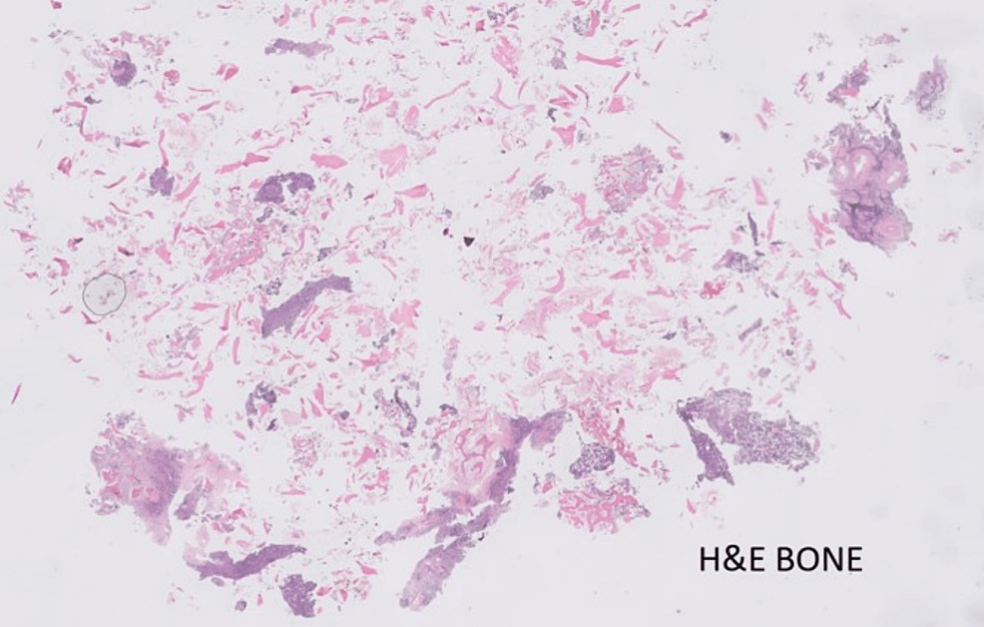
Histopathology - bone

**Figure 3:**
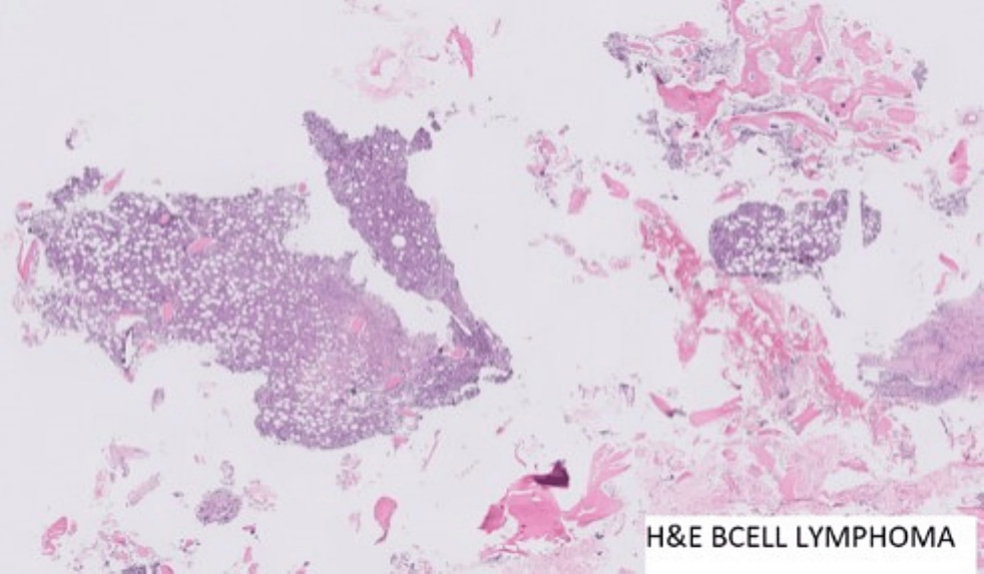
Histopathology - bone marrow biopsy

**Figure 4:**
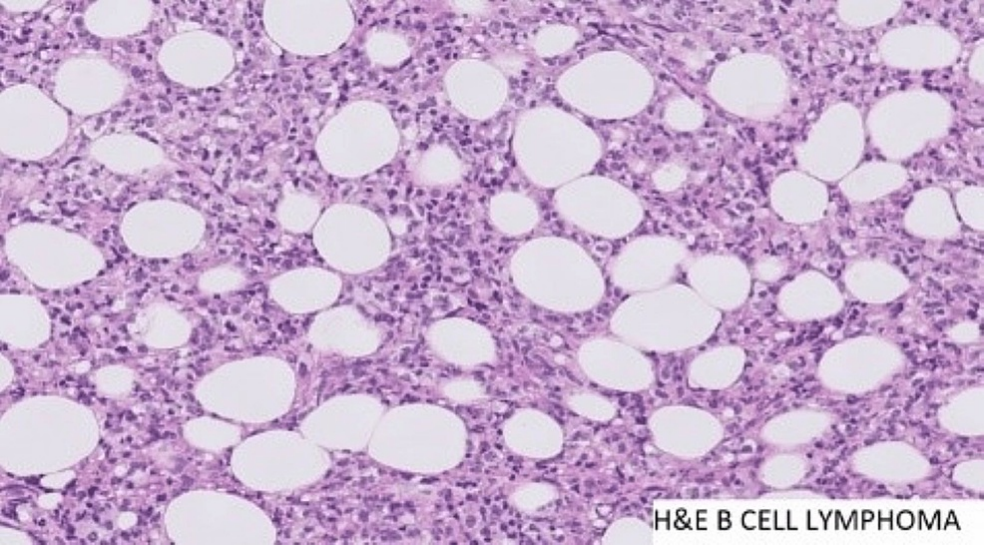
Histopathology - 20× magnification Focally, the cells showed clear cytoplasm. A variable number of histocytes, neutrophils, small lymphocytes, and plasma cells are seen ad mixed. No pseudo rosettes were appreciated. Minimal amount of stroma noted. Frequent mitosis and apoptotic bodies were seen. Desmoplastic reaction with areas of haemorrhage and necrosis was noted. Cells were seen infiltrating into the adjacent soft tissue. Sample was negative for granuloma.

Culture samples were negative. Histopathology (Figures 2-4) showed multiple fragments of bony tissue with focal cellular areas composed of cells arranged in diffuse sheets. Individual cells were uniformly small to medium-sized, round or oval, with scanty cytoplasm, indistinct cytoplasmic borders, and round or irregular nuclei with fine chromatin and inconspicuous nucleoli.

**Figure 5:**
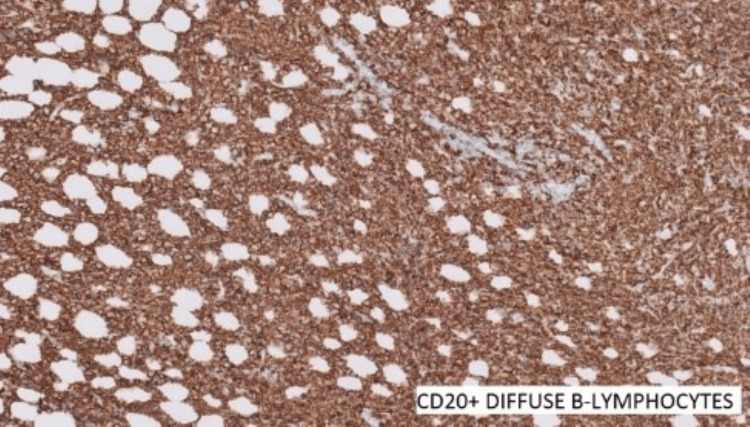
CD20 positive diffuse B lymphocytes

**Figure 6:**
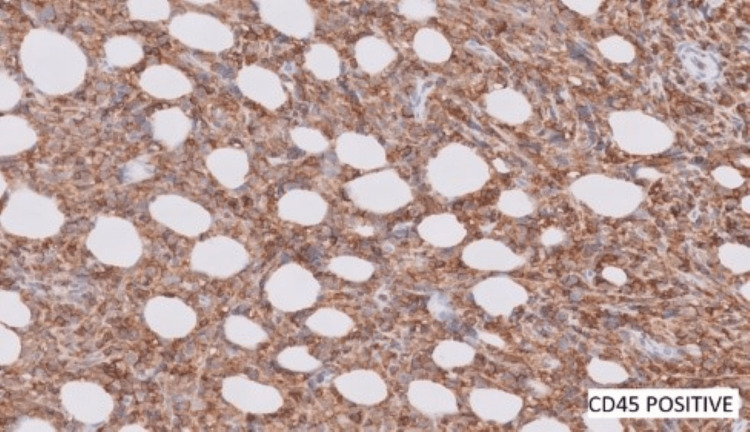
CD45 positive B lymphocytes

**Figure 7:**
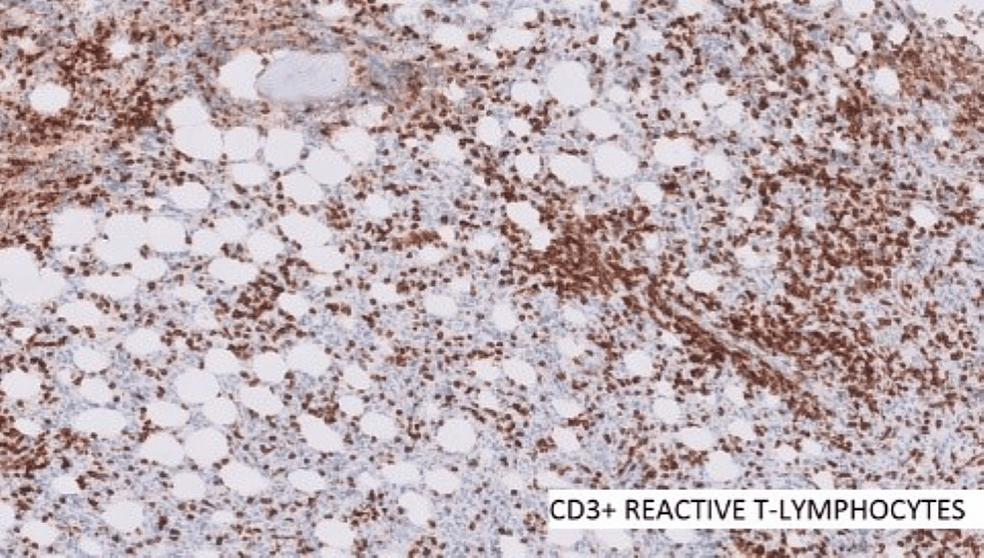
CD3 positive reactive T-lymphocytes

A provisional diagnosis of a B-cell lymphoproliferative lesion was considered. Since B-cell lymphoma had to be ruled out, ancillary tests (immunohistochemistry) were performed (Figures 5-7). It showed neoplastic cells with diffusely strong positivity for CD45 and CD20. Reactive T lymphocytes were highlighted by CD3. Cells were negative for S100, Pan CK, Desmin, CD30, synaptophysin, PAS, and PASD. There were atypical cells expressing heterogeneously with B-cell markers, and immunomorphology could not completely exclude features of lymphoid neoplasms.

Although clinically he had a chronic disease without generalized lymphadenopathy, B-symptoms, pruritis, or other risk factors, and his peripheral smear and lactate hydrogenase (LDH) levels were normal, it was decided to pursue further investigations with the intention of ruling out non-Hodgkin’s lymphoma. He was referred to medical oncology for assessment of visceral involvement.

**Figure 8:**
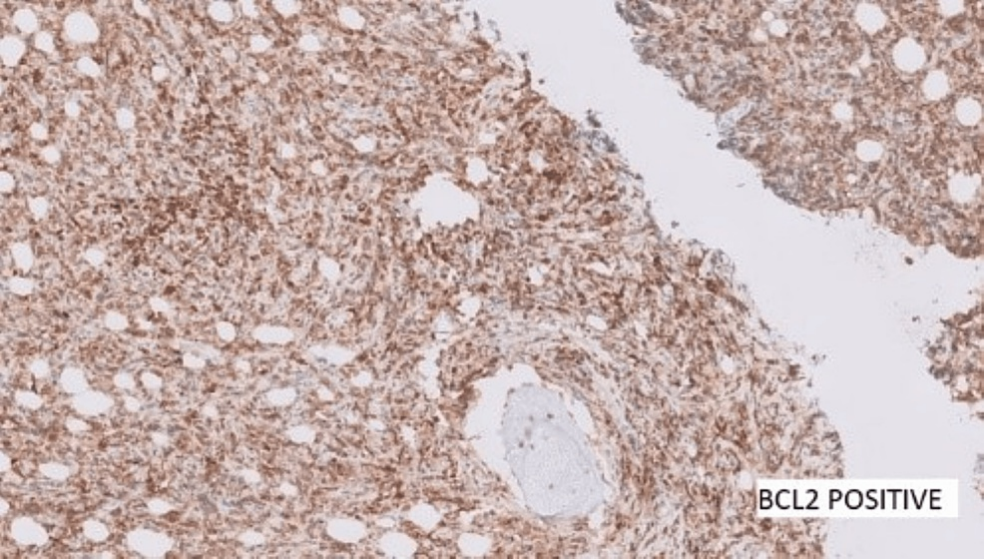
BCL2 positive

**Figure 9:**
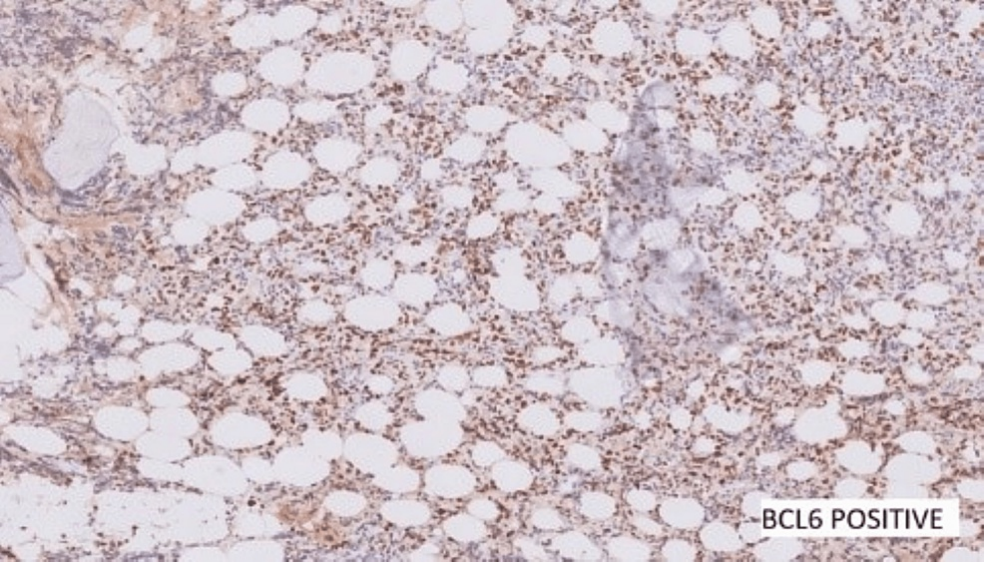
BCL6 positive

**Figure 10:**
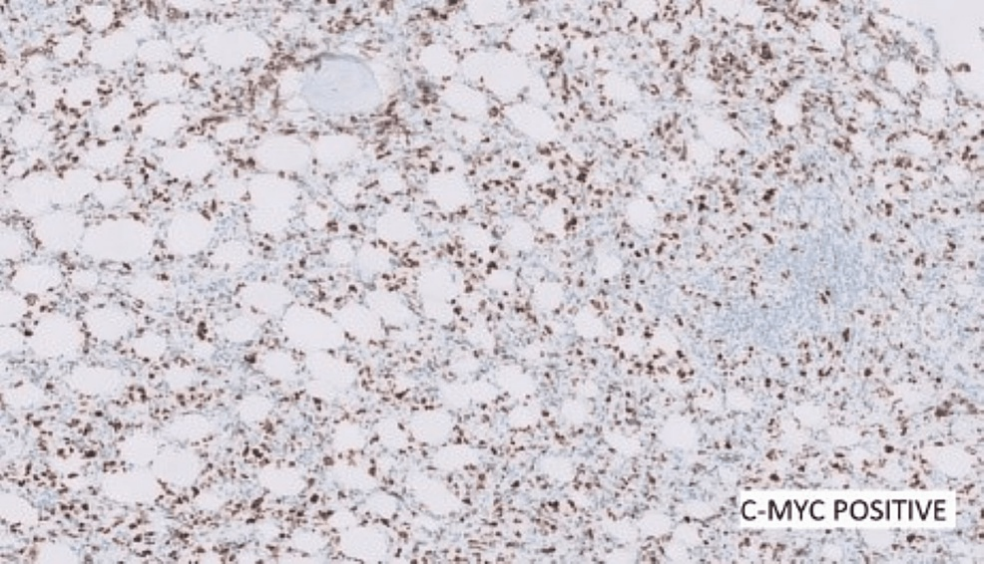
C-MYC positive

**Figure 11:**
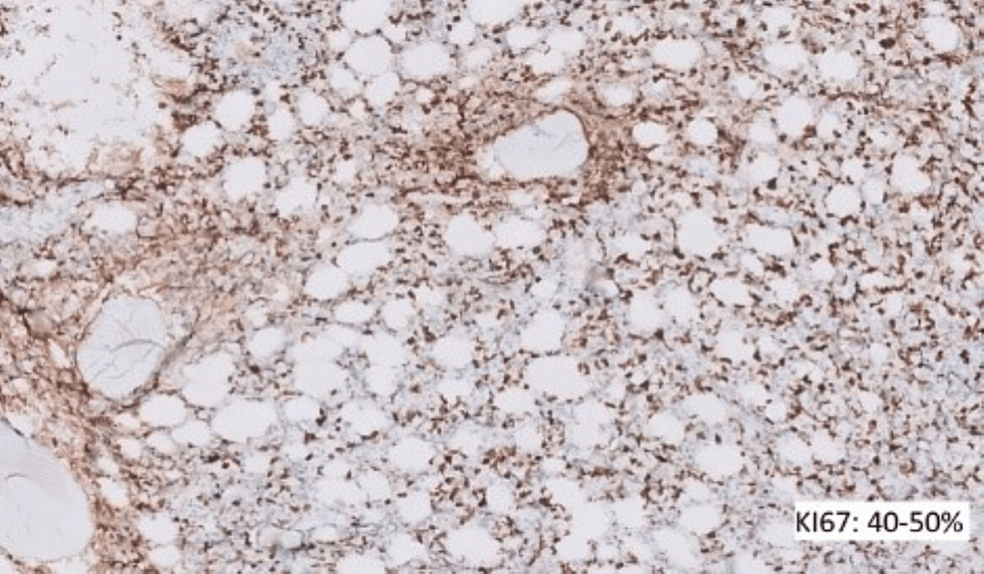
Ki67 - 40-50%

Contrast computed tomography (CT) scans of the chest, pelvis, and abdomen were done. All reported being negative for lymphoma, and no nodes were detected. Further ancillary tests for IHC were performed and showed positive cells for BCL2, BCL6, and c-myc (Figures 8-10) and negative for CD10, MUM1, and TdT. Ki67 was 40-50% (Figure 11).

**Figure 12:**
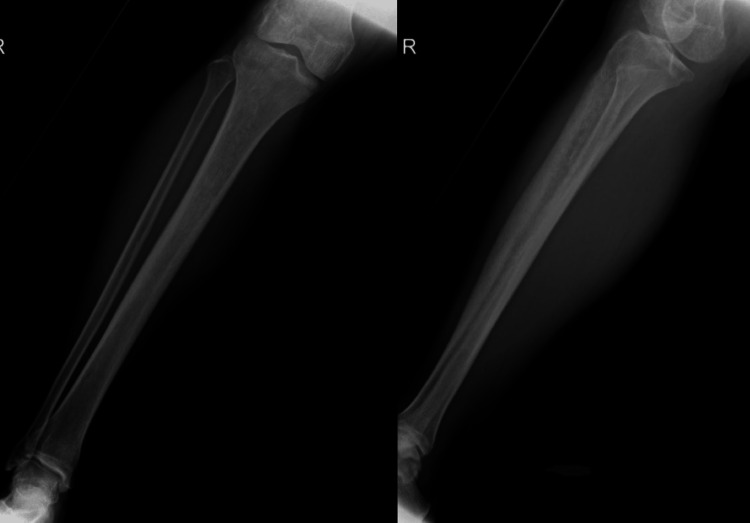
X-ray: AP and lateral views, three months after surgery X-ray showed ill defined, intramedullary, mixed sclero-lytic lesion in meta-diaphysis and proximal tibia with cortical thickening and wide zone of transition. There was no periosteal reaction, joint involvement, or soft tissue enhancement. The sclerotic areas were attributed to calcium phosphate bone cement inserted during surgery.

**Figure 13:**
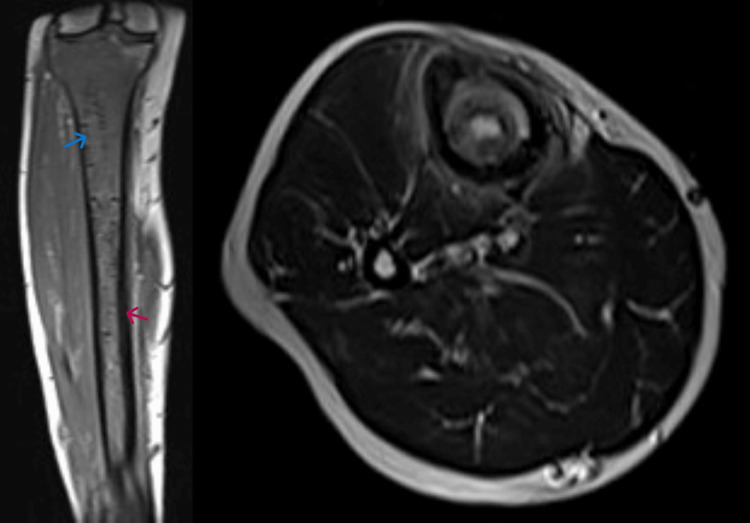
T1 coronal and axial images, three months after surgery Shows hypointense (blue arrow marking deposits of calcium phosphate cement inserted at index surgery) and isointense signals over the proximal to middle third of the shaft of the tibia. Cortical thickening over the middle third shaft could be appreciated (red arrow).

**Figure 14:**
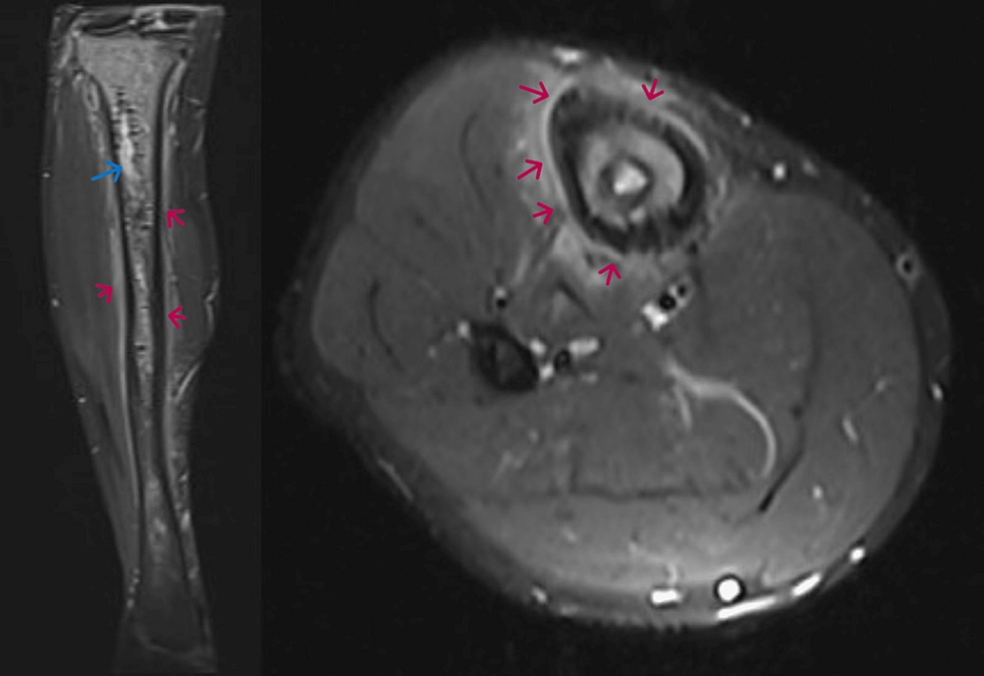
T2 coronal and axial images, three months after surgery The hyperintense signal in the proximal third (blue arrow) could be attributed to the calcium phosphate bone cement inserted at the time of surgery. The periosteal reaction (red arrows) surrounding the bone was probably the result of intramedullary reaming performed at the time of surgery for osteomyelitis.

In the meanwhile, the patient improved symptomatically over the next three months until he had a recurrence of symptoms. X-rays and MRIs were repeated and shown below (Figures 12-14).

In view of the histopathology and IHC features and the recurrence of symptoms within a very short interval after surgery, there was a strong suspicion of a neoplastic disorder. A positron emission tomography (PET) CT scan and biopsy were advised. Since this was not a case of osteomyelitis anymore, the patient was referred to a tertiary-level oncology centre for further management. After a detailed review of the existing records, a CT-guided needle biopsy was performed by an orthopaedic oncologist.

**Figure 15:**
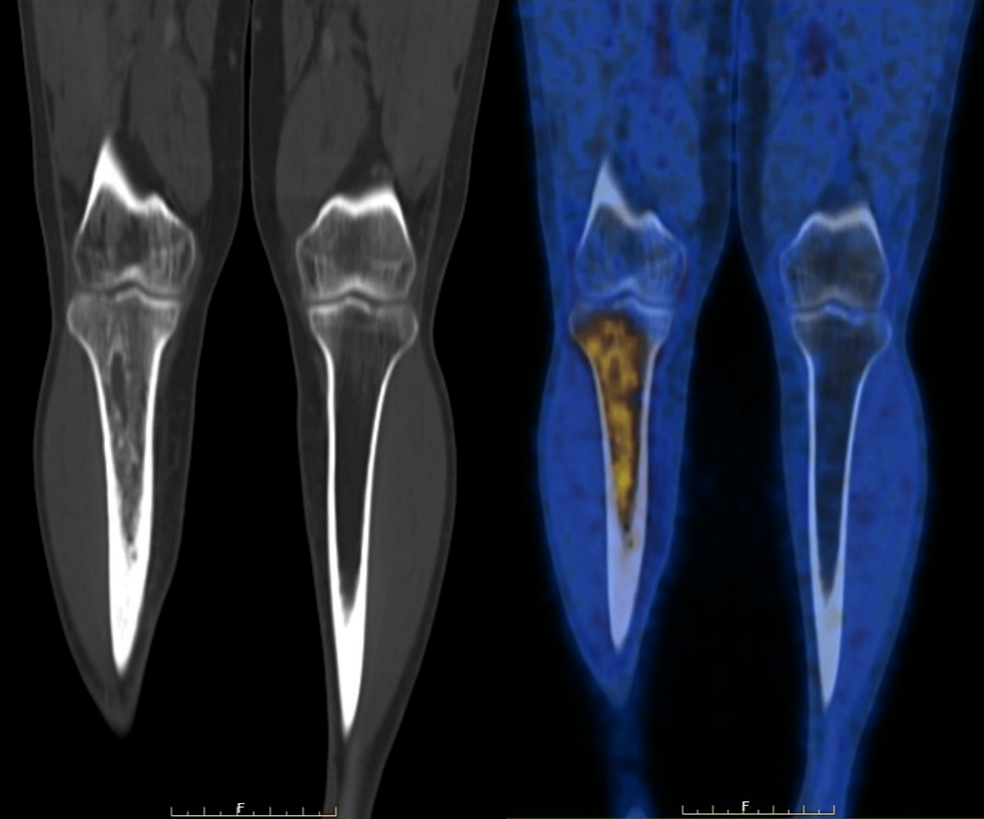
Positron emission tomography FDG avid permeative lytic lesions involving the diaphysis extending to the metaphysis and epiphysis of the right tibia - metabolically active proven primary lymphoma. FDG avid multiple right external iliac, right femoral and multiple lymph nodes above the popliteal fossa (largest measuring 13 mm × 6mm), suggestive of metabolically active lymphomatous deposits (not shown in the above figure) were also noted in this scan.

The IHC report showed cells diffusely positive for CD20 and BCL2 and negative for CD3, with a diagnosis suggestive of non-Hodgkin B-cell lymphoma. He was referred to the medical oncology department for chemotherapy. A PET-CT scan was done (Figure 15).

A definitive diagnosis of extranodal non-Hodgkin B-cell lymphoma, right tibia lesion, Ann Arbour stage II E, was established and treatment initiated. Six cycles of chemotherapy - RCHOP (rituximab/cyclophosphamide/vincristine/prednisolone), were administered over the course of four months.

The PET-CT scan was repeated one month after the completion of chemotherapy. When compared to the previous PET-CT, this scan showed persistence of the lesion with a marginal reduction in the extent of flourodeoxyglucose (FDG) avidity of the permeative lytic lesion involving the diaphysis, extending to the metaphysis and epiphysis of the right tibia, indicating metabolically active residual lymphoma (Deauville score 3). There was metabolic resolution with a reduction in the sizes of the right external iliac, right femoral, and multiple lymph nodes above the right popliteal fossa, and all of them appeared sub-centimetric in size (Deauville score 1). No new metabolically active lymphomatous deposits were noted.

**Figure 16:**
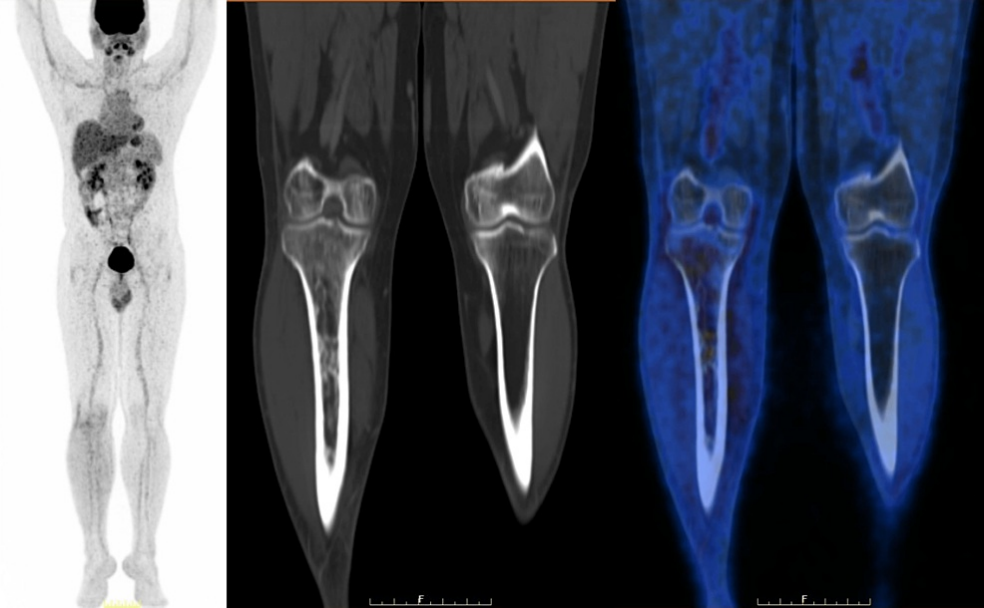
Positron emission tomography, three months after completion of chemotherapy and radiotherapy Metabolic resolution of permeative lytic lesion with intramedullary sclerosis involving diaphysis extending to metaphysis and epiphysis of the right tibia - Status quo in CT, Deauville score 1, post chemo-radiation. Resolution of FDG non-avid lymph nodes over right external iliac, bilateral superficial inguinal, right femoral node and right popliteal fossa were noted.

The case was discussed by the multi-disciplinary tumour board, and it was decided to proceed with consolidation radiation therapy. Three-dimensional conformal RT was started immediately. He received a dose of 45 Grey in 25 fractions over two months and tolerated the treatment well without any major side effects. PET-CT was repeated three months after the completion of radiotherapy (Figure 16). These scans showed the resolution of the lesion.

## Discussion

There are a number of case reports in the literature where the PBL mimicked subacute and chronic osteomyelitis and was treated as osteomyelitis [7-10]. The rarity of the disease and the misleading histopathological and imaging features placed PBL last on the list of our differential diagnoses for a 32-year-old male patient with insidious onset pain, intermittent fever, and diffuse and tender swelling with normal X-ray and non-specific MRI features. However, the histopathological and IHC features pointing to possible B-cell lymphoma, along with the recurrence of symptoms three months post-surgery, prompted us towards a repeat biopsy and further evaluation.

Literature on MRI features in PBL is not forthcoming. Low signal intensity (SI) on T2 weighted spin echo (SE) images, due to large amounts of fibrous tissue, has been mentioned as a feature of PBL [11]. The majority of patients (28 out of 29) in the Heyning et al. [12] series demonstrated isointense soft tissue signal intensity (SI) on T1-weighted sequences and high SI on T2-weighted images. Only one patient demonstrated low SI on T2-weighted images. Cortical involvement was noted in 93% of cases, with cortical breach and soft tissue infiltration in 76%. In 31% of their patients, there were only linear cortical signal abnormalities without substantial soft tissue mass, presenting a very non-aggressive and benign picture. Heyning et al. concluded that MRI features in PBL were not uniform. Mulligan et al. [13] and Krishnan et al. [14] studied the pattern of cortical destruction and used it as a differentiating criterion. Krishnan et al. were of the opinion that a meta-diaphyseal lesion in a patient older than 30 years with marrow signal changes, cortical involvement, and associated soft tissue mass was highly suggestive of PBL. Stiglbauer et al. [11] suggested that there are three features that could be characteristic of PBL: (1) hypointense signals on T2-weighted SE images; (2) tumour location towards the end of the long bones; and (3) a probable tendency towards the involvement of adjacent joints.

Our patient had MRI features consistent with the majority of cases in the Heyning series. He did not have the three characteristics proposed in the Stiglbauer series of cases. The majority of literature on MRI features is rather non-specific, mainly due to a restricted sample size and a focus on the appearance of cortical bone relative to soft tissue extensions.

Although the histopathology report showed B-cell infiltrate positive for CD45 and CD20 markers, a contrast CT of the chest, pelvis, and abdomen did not pick up any abnormality. It can be argued that an FDG-PET scan was indicated at this point in time. The sensitivity, specificity, and accuracy rates for the staging of extranodal lymphomas with PET-CT have been superior (97%, 100%, and 98%, respectively) compared to those of conventional CT imaging (87%, 85%, and 84%) [15].

Primary bone lymphomas are staged as per the Ann Arbour criteria and according to the extent of involvement [16]. The involvement of bone and local or regional lymph nodes will make it stage 1 or 2. Even if there is involvement of the marrow, it is not considered stage 4, unlike other non-Hodgkin lymphomas. This has a direct implication on the prognosis, as the International Prognostic Index (IPI) score depends on the stage of the disease, the LDH levels, and the performance status. Almost all bone lymphomas will be grouped into the low-risk group, with a five-year survival rate of more than 90% [17].

Randomized control trials addressing treatment alternatives are few and far between, considering the rarity of the disease. Current treatment protocols are largely based on recommendations from retrospective studies. Radiotherapy was once considered the treatment of choice. Although local control was achieved, there were high recurrence rates. Currently, there are multiple treatment modalities in place that demonstrate improved survival rates with the combination of chemotherapy and radiation rather than chemotherapy or radiotherapy alone [18].

Our patient was treated with a combination of radiotherapy and chemotherapy. He achieved remission nine months after the initiation of treatment and has been on regular follow-ups since. He was followed up with FDG-PET scans. MRI, a much cheaper test, also shows the extent of the disease (abnormal signal intensities on T1 and T2 weighted images with minimal contrast enhancement) and has proven equally effective as a monitoring tool for response to therapy compared to FDG-PET [19]. However, in our case, since his initial MRI was quite deceptive, it was decided to proceed with FDG-PET as a modality for assessing response to treatment. Besides, PET-CT is a valuable tool here to differentiate active disease from fibrosis.

Traditionally, survival rates have varied from 58% to 75% for bone lymphomas treated with chemotherapy. The addition of rituximab has improved survival by more than 90%. The addition of radiation is a controversial area and has to be individualized based on the end-of-chemotherapy assessment. The incorporation of radiation reduces the chances of local recurrence [20].

## Conclusions

We present a case of PBL that was erroneously diagnosed and treated as subacute osteomyelitis. A biopsy following surgery for sub-acute or chronic osteomyelitis with a picture suspicious of B-cell lymphoma should prompt a widening of the diagnostic net to include investigations to confirm a diagnosis of PBL. One should not hesitate to perform a repeat biopsy under CT guidance. Definitive IHC markers and a supportive PET-CT scan usually clinch the diagnosis of primary bone lymphoma, and the patient can be treated medically with combination chemo-radiotherapy with a good outcome.
